# Chromosome number, sex determination, and meiotic chromosome behavior in the praying mantid *Hierodula membranacea*

**DOI:** 10.1371/journal.pone.0272978

**Published:** 2022-08-12

**Authors:** Leocadia V. Paliulis, Emily L. Stowe, Leila Hashemi, Noemi Pedraza-Aguado, Cynthia Striese, Silke Tulok, Thomas Müller-Reichert, Gunar Fabig

**Affiliations:** 1 Biology Department, Bucknell University, Lewisburg, Pennsylvania, United States of America; 2 Core Facility Cellular Imaging, Faculty of Medicine Carl Gustav Carus, Technische Universität Dresden, Dresden, Germany; 3 Experimental Center, Faculty of Medicine Carl Gustav Carus, Technische Universität Dresden, Dresden, Germany; Virginia Tech, UNITED STATES

## Abstract

Praying mantids are important models for studying a wide range of chromosome behaviors, yet few species of mantids have been characterized chromosomally. Here we show that the praying mantid *Hierodula membranacea* has a chromosome number of 2n = 27, and X_1_X_1_X_2_X_2_ (female): X_1_X_2_Y (male) sex determination. In male meiosis I, the X_1_, X_2_, and Y chromosomes of *H*. *membranacea* form a sex trivalent, with the Y chromosome associating with one spindle pole and the X_1_ and X_2_ chromosomes facing the opposite spindle pole. While it is possible that such a sex trivalent could experience different spindle forces on each side of the trivalent, in *H*. *membranacea* the sex trivalent aligns at the spindle equator with all of the autosomes, and then the sex chromosomes separate in anaphase I simultaneously with the autosomes. With this observation, *H*. *membranacea* can be used as a model system to study the balance of forces acting on a trivalent during meiosis I and analyze the functional importance of chromosome alignment in metaphase as a preparatory step for subsequent correct chromosome segregation.

## Introduction

Praying mantids have been known since the early 20^th^ century as critical species for performing a wide range of chromosome-related studies. Studies of praying mantid chromosomes have provided key insights into chromosome-based sex determination and the evolution of sex determination systems [1], kinetochore orientation [2, 3], chromosome stiffness and condensation [2], chromosome alignment [4], and spindle checkpoint regulation [5, 6]. Previous studies revealed that praying mantids typically have either XX (female): X0 (male) or X_1_X_1_X_2_X_2_ (female): X_1_X_2_Y (male) sex determination [1–3]. Male praying mantids in species with X_1_X_1_X_2_X_2_ (female): X_1_X_2_Y (male) sex determination form a sex trivalent in meiosis I [1–3], and have the Y chromosome facing one spindle pole and the X_1_ and X_2_ chromosomes associating with the opposite spindle pole. Because such systems have seemingly unequal spindle attachments on the trivalent, they offer interesting opportunities for studying the regulation of force balance during cell division.

Nearly all of the previous studies on praying mantid chromosomes were done in fixed, stained cells, and showed that the sex trivalent appeared to align at the center of the spindle with the autosomal bivalents [3, 4]. However, these studies typically used fixation methods that were poor at fixing cellular structures like the spindle. In addition, it is possible that conclusions made from these fixed, stained specimens on chromosome behaviors and alignments could be artefacts of harsh fixation and staining protocols. Only one publication shows images of chromosome behavior in living mantid spermatocytes [5]. Li and Nicklas presented a panel of images taken from a living mantid spermatocyte of the mantid *Tenodera sinensis* [5]. In meiosis I of this species, the sex trivalent appears to align with the autosomes in metaphase I and separates the X_1_ and X_2_ chromosomes from the Y chromosome at the same time as the autosomes in anaphase I. The panel of images in this paper, from a living mantid spermatocyte, hints that the sex trivalent of the praying mantid has many more secrets to reveal about force balance during cell division.

Several species of praying mantids have been studied chromosomally, yet most species remain unstudied. Here, we present data on the chromosome number and sex determination for the previously unstudied praying mantid *Hierodula membranacea*. We show live-cell imaging of spermatocytes to measure the position of the sex trivalent on the spindle. Our work suggests that forces are balanced across the sex trivalent.

## Materials and methods

### Species identification and culture

Living *Hierodula membranacea* (Giant Asian Mantis) males were obtained from InsectSales.com (Port Angeles, Washington, USA), and Mantids & More (Mühlheim am Main, Germany). The authors verified the identification provided by the vendors using information in Vermeersch & Unnahachote [7] and the original species description by Burmeister [8]. Mantids were fed houseflies and crickets according to instructions from the providers.

### DNA barcoding

DNA barcoding was done essentially as described in the Carolina Using DNA Barcodes to Identify and Classify Living Things kit (Carolina 211385). DNA was extracted from leg tissue but instead of manual grinding with a plastic pestle, tissue was vortexed with glass beads (MN Bead Tubes Type A Macherey-Nagel BET0432A) and lysis solution for five minutes. Cytochrome c oxidase subunit 1 was amplified using the primers and PCR beads supplied by Carolina and sequenced at Genewiz using the M13forward and M13reverse primers. Sequence was analyzed using Sequencher v5.4.6 and trimmed to approximately 640 bp. Alignments were produced using ClustalOmega (https://www.ebi.ac.uk/Tools/msa/clustalo/) [9].

### Chromosome squashes

Testes from subadult male *Hierodula membranacea* were fixed in 3:1 ethanol:acetic acid for 10 minutes, then rinsed in distilled water for 30 seconds and placed in a solution of 2.5% orcein in 45% acetic acid (in distilled water) for 5 minutes. Samples were then placed in a droplet of 45% acetic acid on a microscope slide. A coverslip was placed over the testes, and the coverslip was pressed firmly over the slide to squash the testes. Squash samples were observed using a Zeiss inverted phase contrast microscope equipped with a 100X, 1.25 N.A. oil-immersion objective and an Infinity3 Camera (Lumenera). Chromosome squashes occupied multiple focal planes, and the images shown are overlays of multiple focal planes revealing in-focus images of all chromosomes.

### Living cell preparations

Living cell preparations of adult male testes were prepared at room temperature according to the method of Lin et al. [10]. Primary spermatocytes undergoing meiosis were filmed across multiple focal planes using a Zeiss Opton inverted phase contrast microscope as described in Lin et al. [10], or using a Zeiss Observer microscope equipped with a 40X, 0.65 N.A. objective and an Axiocam 503 monochrome camera.

### Quantification of chromosome positioning

The positions of the spindle poles were estimated by the vertex of the cleared areas of each cell, as the spindle excludes phase-dense organelles. A line was drawn between the estimated spindle poles, showing the spindle axis. An additional line was drawn perpendicular to the spindle axis at the midpoint of the line representing the axis, representing the center of the spindle. The distances between the edges of the chromosome facing the pole (estimated kinetochore) and the perpendicular line were measured. The ratio of these distances was then calculated. Similar measurements were done for two autosomes of the same cell.

## Results

### DNA barcoding

The experiments described were performed using *H*. *membranacea* obtained from two providers, one in the USA and the other in Germany. To verify our identification of the mantids from both origins and confirm that the mantids were members of the same species, we performed DNA barcoding analysis on one individual from each source. Sequences of both specimens were submitted to Genbank. The sample obtained in the USA has accession number MZ571202, while the sample obtained from the German provider has accession number MZ571203.

**Fig 1:**
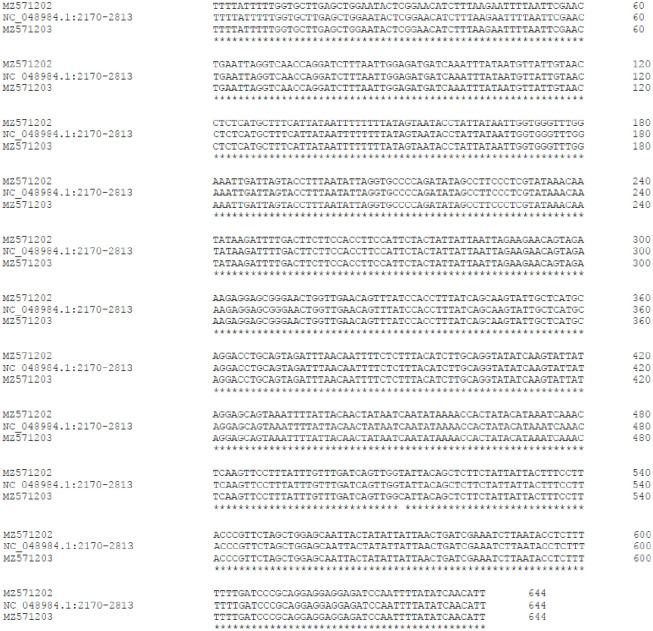
Alignment of nucleotides 2170 to 2813 of NC_048984.1 (complete mitochondrial genome sequence of *H*. *membranacea*) to MZ571202 (isolate from mantid obtained from US provider) and MZ571203 (isolate from mantid obtained from German provider). The MZ571202 isolate was 100% identical to the sequence of NC_048984.1, and the MZ571203 isolate varied at one base and therefore was >99% identical.

The partial Cox1 gene sequences were analyzed via blastn and identified the sequence associated with NC_048984.1, the complete genome sequence of the mitochondrion of *H*. *membranacea*. The sequence corresponding to bases 2170 to 2813 of NC_08984 and the full sequences of MZ571202 and MZ571203 were used in Clustal Omega [9] to create the alignment. While the MZ571202 isolate was 100% identical to the sequence of NC_048984.1, the MZ571203 isolate varied at one base and therefore was >99% identical (Fig 1), consistent with both specimens’ species identification as *Hierodula membranacea*.

### Karyotype analysis

**Fig 2:**
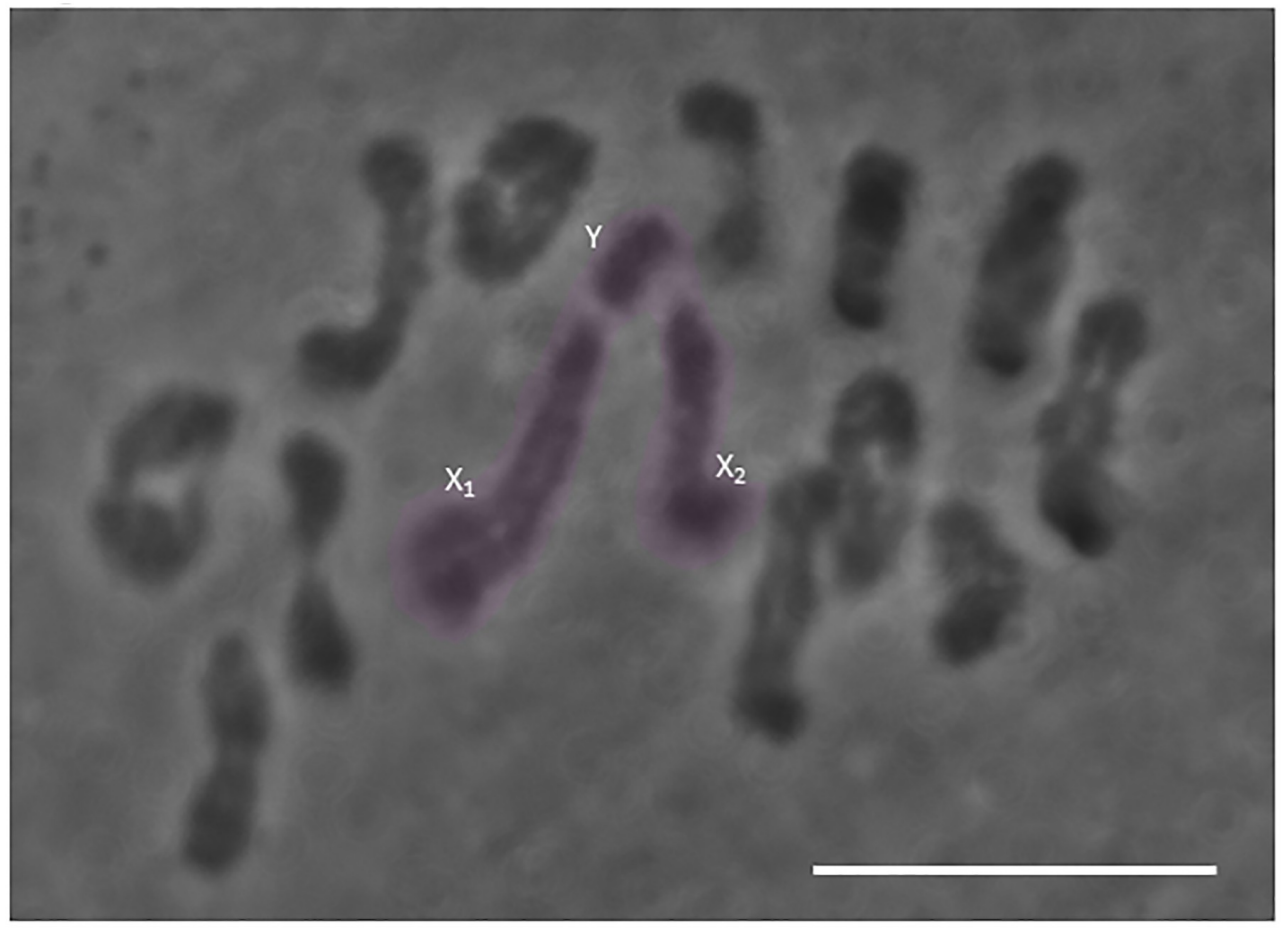
Aceto-orcein squash of a meiosis I spermatocyte in the praying mantis *Hierodula membranacea*. Twelve bivalents and one sex trivalent (highlighted in pink with component sex chromosomes noted) are present in chromosome spreads of this species. Scale bar = 5μm.

Chromosome squashes were constructed using aceto-orcein stained fixed preparations of cells in metaphase I and metaphase II. Preparations from 30 individuals were used to determine the karyotype. Twelve bivalents and one trivalent were present in all spread preparations (Fig 2), indicating that *H*. *membranacea* has a chromosome number of 2n = 27 in males. In male meiosis I, the X_1_, X_2_, and Y chromosomes form a sex trivalent (Fig 2, highlighted in pink).

### Chromosome behavior in meiosis I

**Fig 3:**
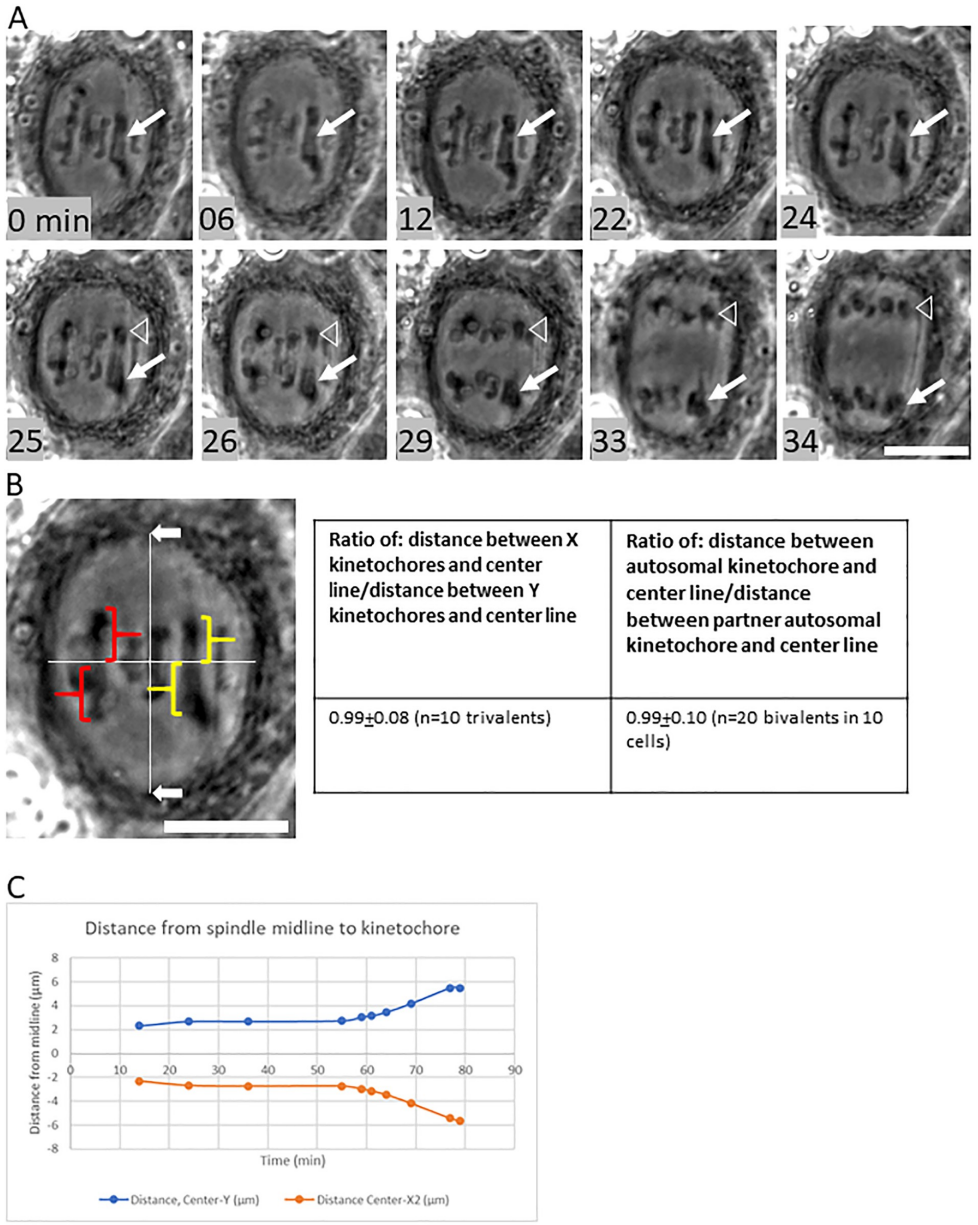
Segregation of chromosomes in meiosis I in the praying mantis *Hierodula membranacea*. **A)** Meiosis I spermatocyte with sex trivalent (arrow). The sex trivalent remains aligned on the metaphase plate with the autosomes (0, 6, 12, 22 min). Anaphase begins (24 min) and the Y chromosome (arrowhead) separates from the X_1_ and X_2_ chromosomes, which move together to the same spindle pole (arrow—26, 29, 33, 34 min). Scale bar = 10μm. **B**) Analysis of the positioning of the trivalent and the autosomes at metaphase I. A line was drawn between the estimated spindle poles (white arrows), showing the spindle axis (left panel). A second line was drawn perpendicular to the spindle axis at the midpoint of the line representing the spindle axis. The distances between the edges of the chromosome facing the pole (estimated kinetochore) and the perpendicular line were measured (yellow braces for the trivalent). The ratio of these distances was then calculated in ten spermatocytes (right panel). Similar calculations were made for two autosomes in the same cell (red). **C**) Graph of position of X_2_ and Y kinetochores of the trivalent shown in A. The trivalent has a balanced alignment at the center of the spindle, with the Y kinetochore maintaining the same distance from the spindle midline as the X_2_ kinetochore.

*H*. *membranacea* has a sex trivalent in meiosis I, which contains an X_1_, an X_2_, and a Y chromosome (Fig 2). The trivalent attaches to the spindle such that the Y chromosome associates with one spindle pole and the X_1_ and X_2_ chromosomes both associate with the opposite spindle pole (Fig 3A). To measure whether the trivalent had a balanced attachment, we estimated the position of the spindle poles by locating the vertices of the cleared region of the cell (the spindle excludes phase-dense organelles like mitochondria, so it is straightforward to estimate the position of poles by looking where the cell is cleared). The distance between the spindle poles was measured and then a line perpendicular to the line between poles was drawn. In all cells that we measured, the X_1_ and X_2_ chromosomes of the trivalent were positioned adjacent to one another, so the level of balance of the chromosome was determined by measuring the distance between the line perpendicular to the spindle axis and the X kinetochores, then measuring the distance between the line perpendicular to the spindle axis and the Y kinetochores, and calculating the ratio between the two (Fig 3B). Because the X_1_ and X_2_ chromosomes in *H*. *membranacea* are submetacentric (i.e. chromosomes have two arms but centromeres are not equidistant from both chromosome ends), free (unstretched) chromosome arms form an angle with the remainder of the trivalent at the X_1_ and X_2_ kinetochores. The position of the kinetochore is the position on the X chromosomes of the trivalent where the trivalent forms an angle. In all cells measured (n = 10), the trivalent had a balanced alignment, giving a ratio of 0.99 ± 0.08, showing approximately 50% of its length on one side of the spindle midline and 50% on the other side of the midline. This balanced alignment of the trivalent was approximately equivalent to our observations of autosomal bivalents, as the ratio of the distance between the line perpendicular to the spindle axis and each kinetochore was 0.99±.01 (measured in two autosomes in each of the ten cells; Fig 3B).

At the onset of anaphase I, homologous autosomes moved towards their associated spindle poles (Fig 3A). At the same time (onset of anaphase I), the chromosomes of the sex trivalent separated, with the X_1_ and X_2_ chromosomes moving together toward one spindle pole and the Y chromosome moving toward the opposite spindle pole (Fig 3C). There was no delay in anaphase separation of the sex trivalent relative to separation of the autosomes. We have observed progress through anaphase I in five spermatocytes. In all cases, the trivalents appear to segregate with the autosomes without delay.

## Discussion

This is the first description of the chromosome number and sex-determination mechanism in *H*. *membranacea*. The chromosomes of other members of the genus *Hierodula* have been characterized by Oguma [11] and Asana [12]. All members of the genus *Hierodula* that have been described have the same chromosome number and sex trivalent in males. Based on previous studies of praying mantid chromosomes, we deduced a chromosome number of 2n = 28 in females with X_1_X_2_Y (male)/X_1_X_1_X_2_X_2_ (female) sex determination for this species [2].

### Importance of the alignment of the sex trivalent in metaphase I

Chromosomes must be positioned correctly in metaphase to ensure correct chromosome distribution in anaphase. All chromosomes must attach to the spindle, with one kinetochore facing each spindle pole. This bipolar attachment guarantees that partner chromosomes will separate from one another in anaphase. In metaphase, after forming bipolar attachments, chromosomes of nearly all organisms align in the center of the spindle forming the metaphase plate. It is possible that the alignment of chromosomes in the center of the spindle in metaphase may be simply a consequence of a “tug of war,” or a balance of forces that the chromosome is experiencing as a result of a bipolar attachment, with both sides of the chromosome under equal tension [13]. The positioning of chromosomes at the center of the spindle could be essential for guaranteeing correct distribution in anaphase. However, the metaphase alignment of chromosomes at the center of the spindle is not essential for correct chromosome distribution in every anaphase, as correct distribution can happen when chromosomes have bipolar attachments but are not aligned on the metaphase plate [4, 14, 15]. In fact, some rare systems like the meiosis I spermatocytes of the flatworm *Mesostoma ehrenbergii* do not form a metaphase plate, and chromosomes have broad oscillations from pole to pole through anaphase onset [15]. In contrast, failure of chromosomes to align on the metaphase plate leads to aneuploidy and cell death in many cell types [14]. While correct distribution can happen in the absence of alignment, because most organisms do align chromosomes on a metaphase plate, it appears that this is the preferred cytological state for correct chromosome distribution in anaphase in most organisms.

Sex trivalents are evidence for the functional importance of metaphase alignment of chromosomes. Because a single Y kinetochore associates with one spindle pole, while the two other X kinetochores associate with the opposite pole, it is not unreasonable to assume that the forces exerted on the spindle would be unbalanced, and the trivalent would be shifted towards the pole associated with the two X kinetochores. In our images of living cells, the sex trivalent shows a balanced alignment in metaphase I, aligning with all of the autosomes on the spindle. This could be achieved by a balance in the number of microtubules attached to the kinetochores.

Nicklas and Arana [4] examined the alignment of mantid sex trivalents in fixed meiosis I spermatocytes of four species of praying mantids. In this study, we show that sex trivalents align on the metaphase plate in meiosis I in living spermatocytes in this previously-unstudied species with the autosomes. Contributing an additional system to the data on trivalent alignment, our work supports the hypothesis of Nicklas and Arana that congression to the spindle equator is important for correct chromosome segregation [4].

Here we observed that the sex trivalent segregates Y from X_1_ and X_2_ at the same time as the autosomal bivalents separate in early anaphase I. This suggests that release of chromosome cohesion in autosomes and sex chromosomes is regulated by the same rules in anaphase I in *H*. *membranacea*. Interestingly, this behavior contrasts from the behavior of a sex trivalent in a different system. The cellar spider *Pholcus phalangioides* appears to initiate separation of homologous autosomes from one another prior to separation of the sex trivalent, i.e. the separation of X_1_ and X_2_ from Y is delayed in this system [16]. While some phase-dense connections do appear to connect the separating sex chromosomes at some time points in our cells (Fig 3A), they do not appear to delay complete separation of the sex chromosomes as is observed in *Pholcus phalangioides*. It is not clear why there are differences in chromosome segregation in these two systems with sex trivalents, but a comparative analysis of these systems could provide insight into how chromosome cohesion is released in anaphase in general.

## Conclusion

Our results reveal a chromosome number of 2n = 27 and an X_1_X_1_X_2_X_2_ (female) X_1_X_2_Y (male) sex-determination mechanism for the praying mantid *Hierodula membranacea*. We also show that the sex chromosomes form a trivalent in male meiosis I, and that this trivalent aligns on the metaphase I plate. The position of the trivalent indicates that the cell regulates alignment of all chromosomes in metaphase, supporting the hypothesis of Nicklas and Arana [4] that congression of chromosomes to the spindle equator in metaphase is important for subsequent chromosome segregation.

## Supporting information

S1 Video Metaphase I-Anaphase I in a Hierodula membranacea spermatocyte. Primary spermatocyte of *Hierodula membranacea*. The sex trivalent is labeled in the initial frame, showing X_1_, X_2_, and Y (bar = 10μm). The cell periodically drifts out of focus, and the trivalent is re-labeled at the 10:43 time stamp and the 11:14 timestamp, when anaphase has started and the X_1_ and X_2_ chromosomes are separating from the Y chromosome.
(MP4)

## References

[1] White MJD. The evolution of the sex chromosomes. J Genet. 1941;42:173.

[2] Hughes-Schrader S. The chromosomes of mantids (Orthoptera; Manteidae) in relation to taxonomy. Chromosoma. 1950;4:1–55. doi: 10.1007/BF00325766 14812633

[3] Hughes-Schrader S. Polarization, kinetochore movements, and bivalent structure in the meiosis of male mantids. Biol Bull. 1943;85:265–300.

[4] Nicklas RB, Arana P. Evolution and the meaning of metaphase. J Cell Sci. 1992;102: 681–690. doi: 10.1242/jcs.102.4.681 1429886

[5] Li X, Nicklas RB. Mitotic forces control a cell-cycle checkpoint. Nature. 1995;373:630–632. doi: 10.1038/373630a0 7854422

[6] Li X, Nicklas RB. Tension-sensitive kinetochore phosphorylation and the chromosome distribution checkpoint in praying mantid spermatocytes. J Cell Sci. 1997;110: 537–545. doi: 10.1242/jcs.110.5.537 9092936

[7] Vermeersch X, Unnahachote T. Hierodula confusa sp. nov., a new species of Hierodula Burmeister, 1838 (Mantodea: Mantidae: Hierodulinae: Hierodulini). Belg J Entomol. 2020;103: 1–13.

[8] Burmeister H.C. Handbuch der Entomologie. Berlin: Ensiln; 1838.

[9] Fast, scalable generation of high-quality protein multiple sequence alignments using Clustal Omega. Mol Syst Biol. 2011;7:539. doi: 10.1038/msb.2011.75 21988835 PMC3261699

[10] Lin NKH, Nance R, Szybist J, Cheville A, Paliulis LV. Micromanipulation of Chromosomes in Insect Spermatocytes. J Vis Exp JoVE. 2018;(140). doi: 10.3791/57359 30394368 PMC6235582

[11] Oguma K. Karyotype and Phylogeny of the Mantis. La Kromosomo. 1946;1: 1–5.

[12] Asana JJ. Studies on the chromosomes of Indian Orthoptera. IV: The idiochromosomes of Hierodula sp. Curr Sci. 1934;2:244–245.

[13] Fabig G, Kiewisz R, Lindow N, Powers JA, Cota V, Quintanilla LJ, et al. Male meiotic spindle features that efficiently segregate paired and lagging chromosomes. eLife. 2020 Mar 10;9:e50988. doi: 10.7554/eLife.50988 32149606 PMC7101234

[14] Czechanski A, Kim H, Byers C, Greenstein I, Stumpff J, Reinholdt LG. Kif18a is specifically required for mitotic progression during germ line development. Dev Biol. 2015;402: 253–62. doi: 10.1016/j.ydbio.2015.03.011 25824710 PMC4450139

[15] Ferraro-Gideon J, Hoang C, Forer A. *Mesostoma ehrenbergii* spermatocytes–A unique and advantageous cell for studying meiosis. Cell Biol Int. 2013;37: 892–898. doi: 10.1002/cbin.10130 23686688

[16] Král J, Musilová J, St’áhlavský F, Rezác M, Akan Z, Edwards RL, et al. Evolution of the karyotype and sex chromosome systems in basal clades of araneomorph spiders (Araneae: Araneomorphae). Chromosome Res. 2006;14: 859–80. doi: 10.1007/s10577-006-1095-9 17195053

